# Clinical effects of cyclosporine A on reperfusion injury in myocardial infarction: a meta-analysis of randomized controlled trials

**DOI:** 10.1186/s40064-016-2751-y

**Published:** 2016-07-19

**Authors:** Chen Yingzhong, Cai lin, Wang Chunbin

**Affiliations:** Cardiovascular Disease Research Institute, The Third People’s Hospital of Chengdu, The Second Affiliated Chengdu Clinical College of Chongqing Medical University, Sichuan, China; Department of Cardiology, The Third People’s Hospital of Chengdu, Chengdu, 610031 Sichuan China

**Keywords:** Myocardial infarction, Cyclosporine A, Drug therapy

## Abstract

Reperfusion therapy is the most crucial strategy for rescuing ischemic myocardium and reducing infarction size. Cyclosporine A (CsA) can protect against reperfusion-induced myocardial necrosis. However, the clinical effects of CsA on myocardial infarction (MI) remain uncertain. This study investigated the effects of CsA on reperfusion injury (RI) in MI. We searched for and included articles regarding randomized controlled trials investigating the effect of CsA in patients with MI from PubMed, EMBASE, and Cochrane Library databases for an analysis. We then performed quality assessment, subgroup, sensitivity, and publication bias analyses. Of the 277 potentially relevant articles retrieved from the databases, only five were eligible for our meta-analysis. Compared with the placebos used in these studies, CsA did not reduce all-cause mortality [rate ratio (RR) 1.10, 95 % confidence interval (CI) 0.75–1.61; *P* = 0.533; *I*^2^ = 0 %) or adverse clinical events (RR 1.0, 95 % CI 0.89–1.13; *P* = 0.381; *I*^2^ = 6.5 %). In the CsA treatment groups, improvement in left ventricular ejection fraction (weighted mean difference = 1.91; 95 % CI 0.89, 2.92; *P* = 0.064) and reduction in MI size (standard mean difference = −0.41, 95 % CI −0.84 to 0.02; *P* = 0.519; *I*^2^ = 0.0 %) were minimal. The current meta-analysis indicates that CsA treatment does not reduce all-cause mortality and adverse clinical events in MI and that CsA may not have significant clinical effects on RI in MI.

## Background

Myocardial infarction (MI) is a common disabling disease worldwide; in 2009, approximately 683,000 patients were discharged from US hospitals with a diagnosis of acute coronary syndrome. Over the past decade, community incidence rates of ST-elevation MI (STEMI) have decreased whereas those of non-STEMI have increased (O’Gara et al. 2013). In addition, in-hospital (approximately 5–6 %) and 1-year (approximately 7–18 %) mortality rates of STEMI have decreased significantly in association with a substantial increase in the frequency of care including guideline-directed medical therapy and interventions (defect-free care) (O’Gara et al. 2013). The rates of death, heart failure, and recurrent ischemic events occurring in the first year after MI remained excessively high in this high-risk population. Reperfusion therapy is the most crucial strategy for rescuing ischemic myocardium and reducing infarction size (Hausenloy and Yellon 2013). Although numerous advances have been made in developing methods for reopening implicated coronary arteries and preventing reocclusion, there is no specific treatment targeting myocardial reperfusion injury (RI), a paradoxical form of myocardial damage occurring because of the restoration of vessel patency (Heusch 2015).

Mitochondrial dysfunction is a major factor leading to the loss of cardiomyocyte function and viability (Gao et al. 2015). The mitochondrial permeability transition pore (mPTP) is a key feature of cardiac cell death in ischemia–RI (I/R). Two molecular pathways, RISK [PI3K–pAkt–pERK–GSK3β cascade] and SAFE [JNK–STAT3 cascade], inhibit mPTP opening, and strategies for activating these pathways can facilitate reducing RI (Santos-Gallego et al. 2016). The mPTP blocker cyclosporine A (CsA) can protect against reperfusion-induced myocardial necrosis and acute coronary artery permeabilization-related complications (Monassier et al. 2015). An animal study-based meta-analysis indicated that CsA may reduce MI size (Lim et al. 2012). However, the clinical effects of CsA on MI remain largely unknown. A small-scale pilot study reported that compared with a placebo, CsA administration at the time of reperfusion is strongly associated with a smaller MI, according to some measures (Piot et al. 2008). However, another small-scale study reported that CsA treatment does not have beneficial effects on either MI size or other clinical outcomes (Ghaffari et al. 2013). The most clinically relevant indicators of CsA treatment efficacy are reduced mortality and morbidity rates; however, these indicators generally require large samples and long follow-up periods. All randomized controlled trials (RCTs) for CsA have used smaller samples and shorter follow-up periods; thus they have not reported substantial differences in clinical events. Therefore, to clarify the efficacy of CsA therapy for MI, we performed a meta-analysis of relevant placebo-controlled RCTs for CsA treatment of RI in MI.

## Methods

This meta-analysis was performed according to the Preferred Reporting Items for Systematic Reviews and Meta-Analyses (PRISMA) guidelines (Moher et al. 2009).

### Literature search strategy

Two reviewers systematically searched for relevant studies in PubMed, EMBASE, and Cochrane Library databases from any date until February 2016. The search was restricted to articles published in English. The search terms are listed in “Appendix”. We also performed backward snowballing to obtain potentially relevant articles from the reference lists of retrieved RCTs and review articles.

### Study selection

Titles and abstracts of all retrieved articles were independently analyzed by two reviewers who excluded any obviously irrelevant studies. The eligibility of the remaining articles was further assessed using full-text evaluation by the same reviewers. Disagreements between the reviewers were resolved through discussion. Studies were included if they fulfilled the following criteria: (1) they were RCTs, (2) they involved patients with MI, and (3) they involved CsA treatment.

### Data extraction and assessment of risk of bias

Two reviewers independently extracted relevant data from the included articles; a third reviewer repeatedly supervised the review process and resolved disagreements through discussion. The following characteristics of included studies were extracted: title, first author, publication year, journal, country, corresponding address, study design, and inclusion and exclusion criteria. If several articles reported the same study, the one with the most complete data was included in our meta-analysis.

Risk of bias for the included RCTs was independently evaluated by two reviewers by using the Cochrane risk of bias tool (Higgins et al. 2011). Disagreements were resolved through discussion. The quality evaluation was judged on random sequence generation, allocation concealment, blinding of participants and personnel, blinding of outcome assessment, incomplete outcome data, selective reporting, and other sources of bias.

### Statistical analysis

All statistical analyses were completed using Stata 12.0 (StataCorp. 2011. Stata Statistical Software: Release 12; StataCorp LP, College Station, TX, USA) and RevMan software (version 5.3; Cochrane Collaboration, Oxford, UK). Heterogeneity was evaluated using the Chi squared test (*P* ≤ 0.10 indicated significant heterogeneity) and *I*^2^ test (*I*^2^ > 50 % indicated significant heterogeneity). For categorical variables, we calculated the rate ratio (RR) as well as the corresponding 95 % confidence intervals (CIs) for the outcome variables of interest. For continuous data, mean differences (MDs) with corresponding 95 % CIs were calculated. If there was no significant heterogeneity among the included studies, an inverse variance fixed-effect model was used; otherwise, a random-effects model was used. Sensitivity analysis was performed to identify the stability of statistical results through individually excluding each study from the analysis. In addition, publication bias was evaluated using funnel plots and Egger’s test. Statistical significance was defined as *P* < 0.05.

## Results

### Eligible studies

**Fig. 1:**
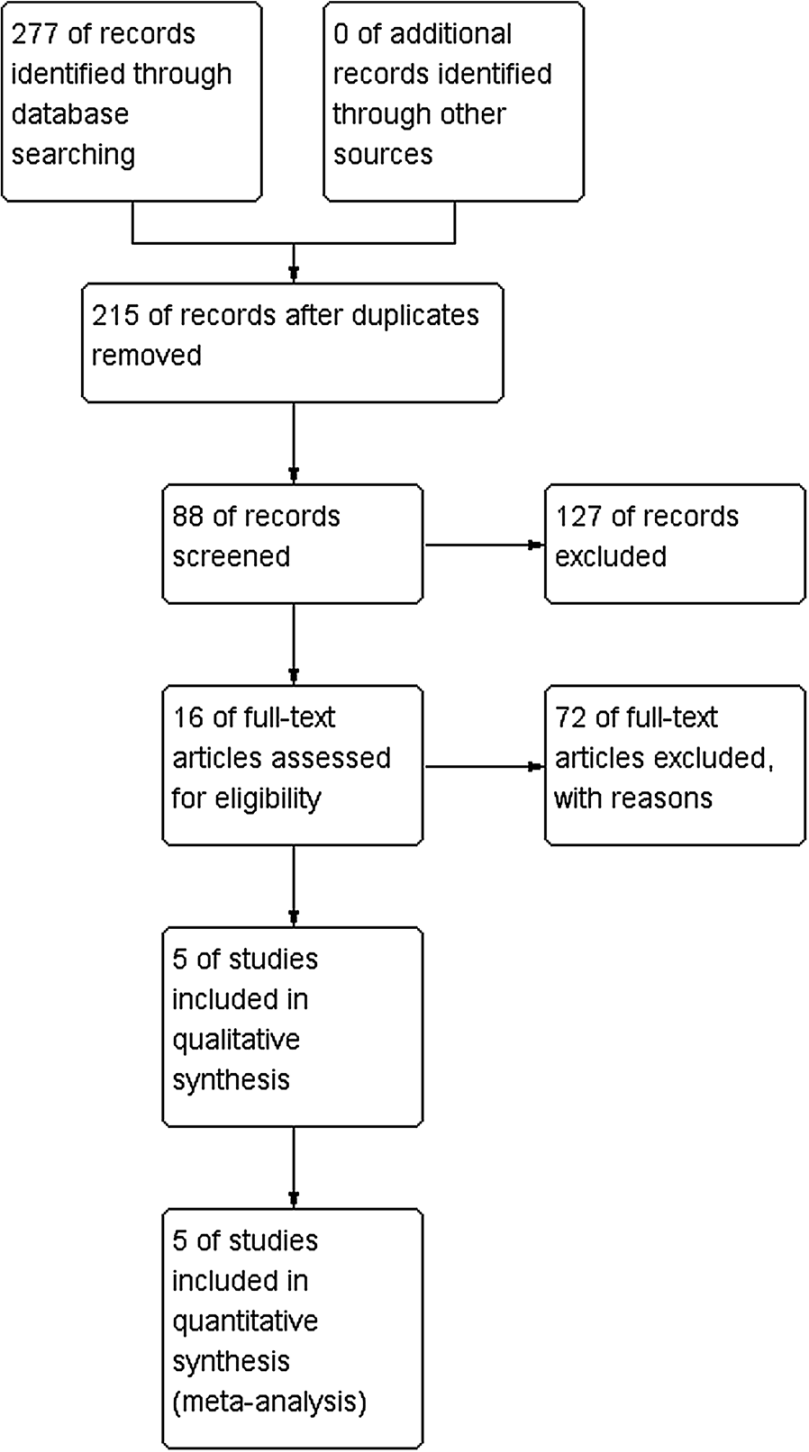
PRISMA flowchart of the study selection process

In total, our database search yielded 277 potentially relevant articles, of which 215 remained after duplicates were removed. After the titles and abstracts of the remaining articles were examined, 127 were excluded and the remaining 88 were screened further. After a full-text evaluation, 11 articles remained, six of which were excluded for the following reasons: four were duplicated publications, one was a review, and one did not involve humans. Finally, five studies were deemed eligible for our meta-analysis. The process of the literature search and reasons for exclusion are presented in Fig. 1.

### Characteristics of selected studies

The five included articles were double-blinded RCTs, in which CsA was administered as an intravenous bolus dose (2.5 mg/kg). The average patient age varied from 58 to 67 years. The major characteristics of the selected studies are listed in Table 1.

**Table 1:** Characteristics of the study population

Study	Design	Participants	Cyclosporine method	Cyclosporine dosage (mg/kg)	Follow-up	Age, year	Number of subjects
Ottani2016	Prospective, multicenter, randomized, controlled, double-blind	ST-segment elevation MI	Intravenous bolus	2.5	4 days 6 months	62.5 ± 12.4	207
Cung2015	Prospective,multicenter, randomized, controlled, double-blind	Patients with acute Anterior STEMI Accepting PCI	Intravenous bolus	2.5	12 months	60.4 ± 13.1	474
Ghaffari2013	Randomized,placebo-controlled, double-blinded	Patients with acute Anterior STEMI Receiving TLT	Intravenous bolus	2.5	1 day 6 months	64.0 ± 11.2	50
Mewton 2010	Prospective, multicenter, randomized, controlled, single-blind	Patients with AMI Accepting PCI	Intravenous bolus	2.5	5 days 6 months	60 ± 10	15
Piot2008	Prospective, multicenter, randomized, controlled, single-blind	Patients with AMI Accepting PCI	Intravenous bolus	2.5	2 days 3 months	58 ± 2	30

### Data quality

**Fig. 2:**
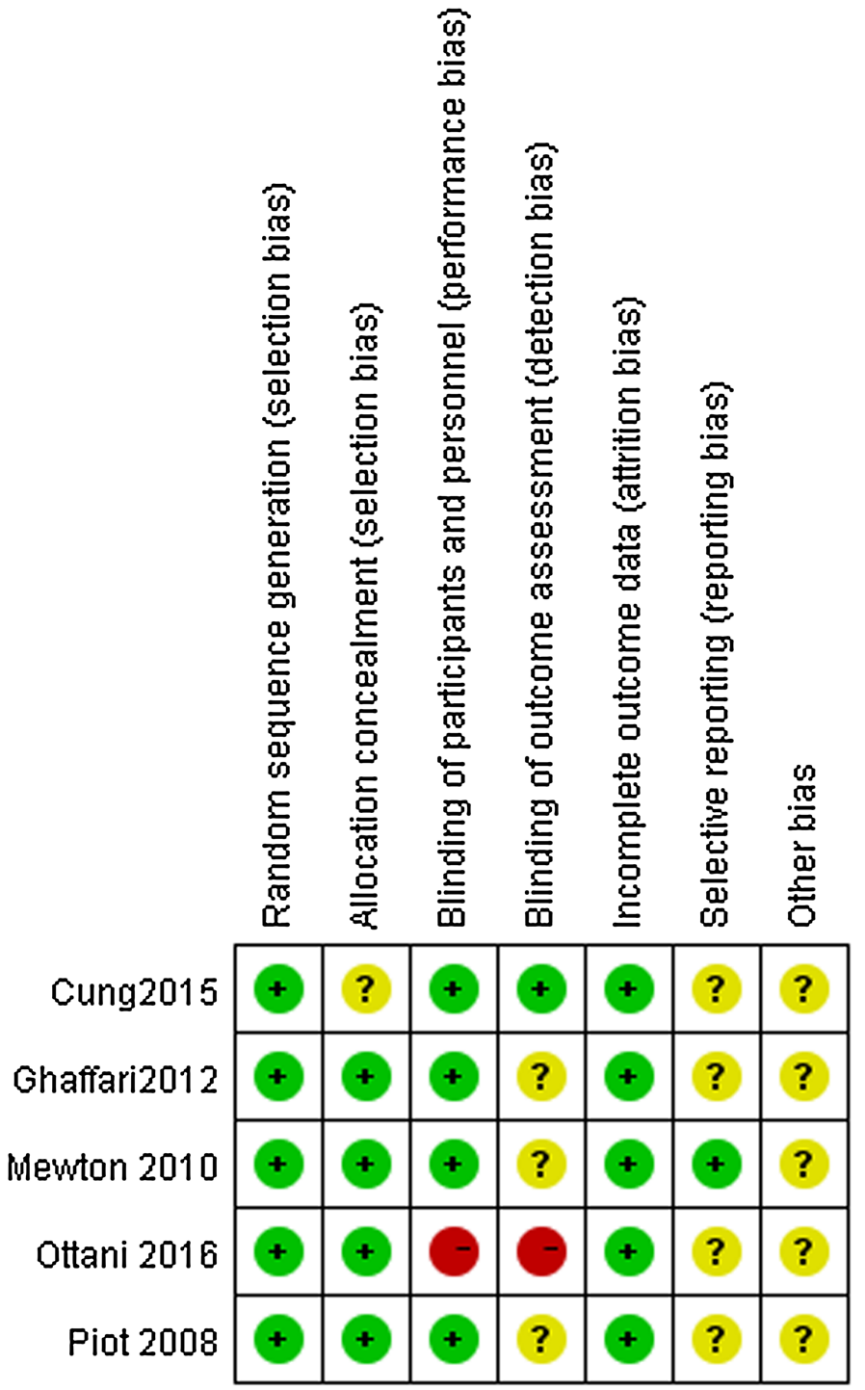
Risk of bias summary

**Fig. 3:**
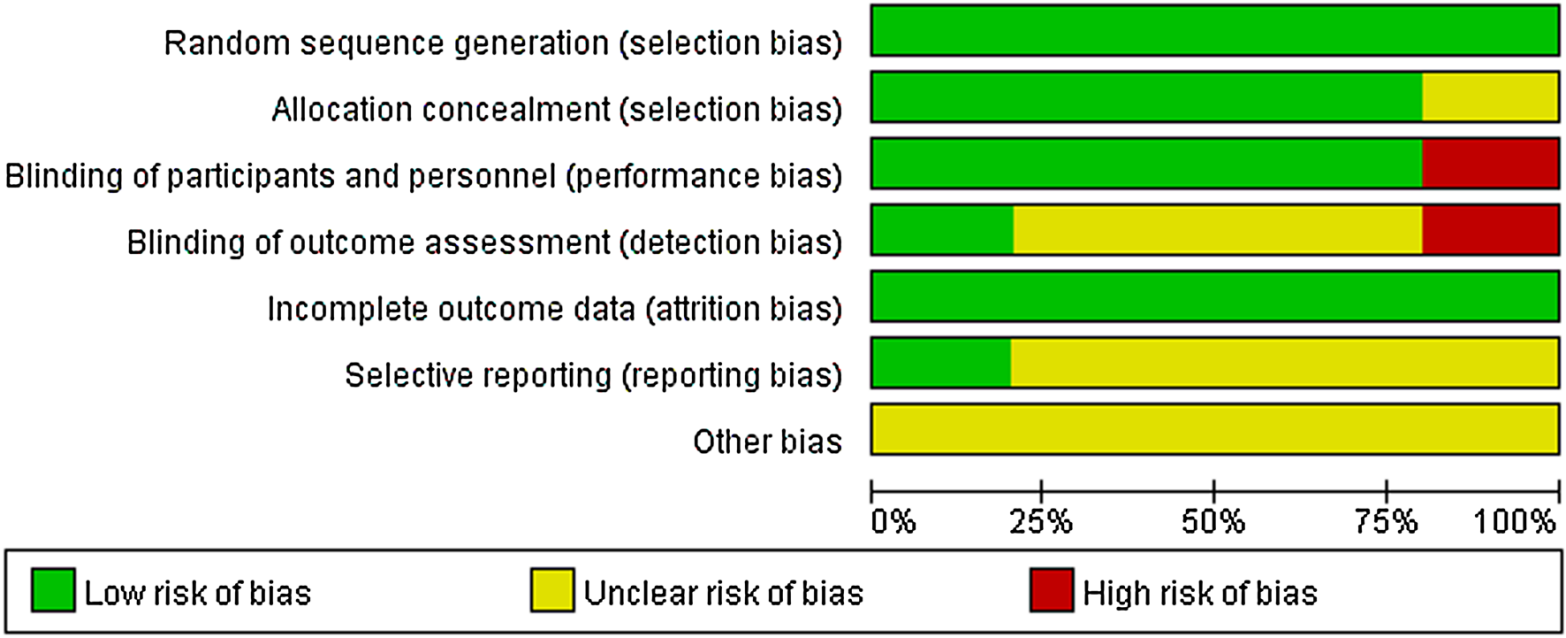
Risk of bias graph

The quality scores of the trials varied from 3 to 5. All included RCTs were randomized, prospective, placebo-controlled, and double-blinded (Figs. 2, 3).

### Efficacy outcomes

**Fig. 4:**
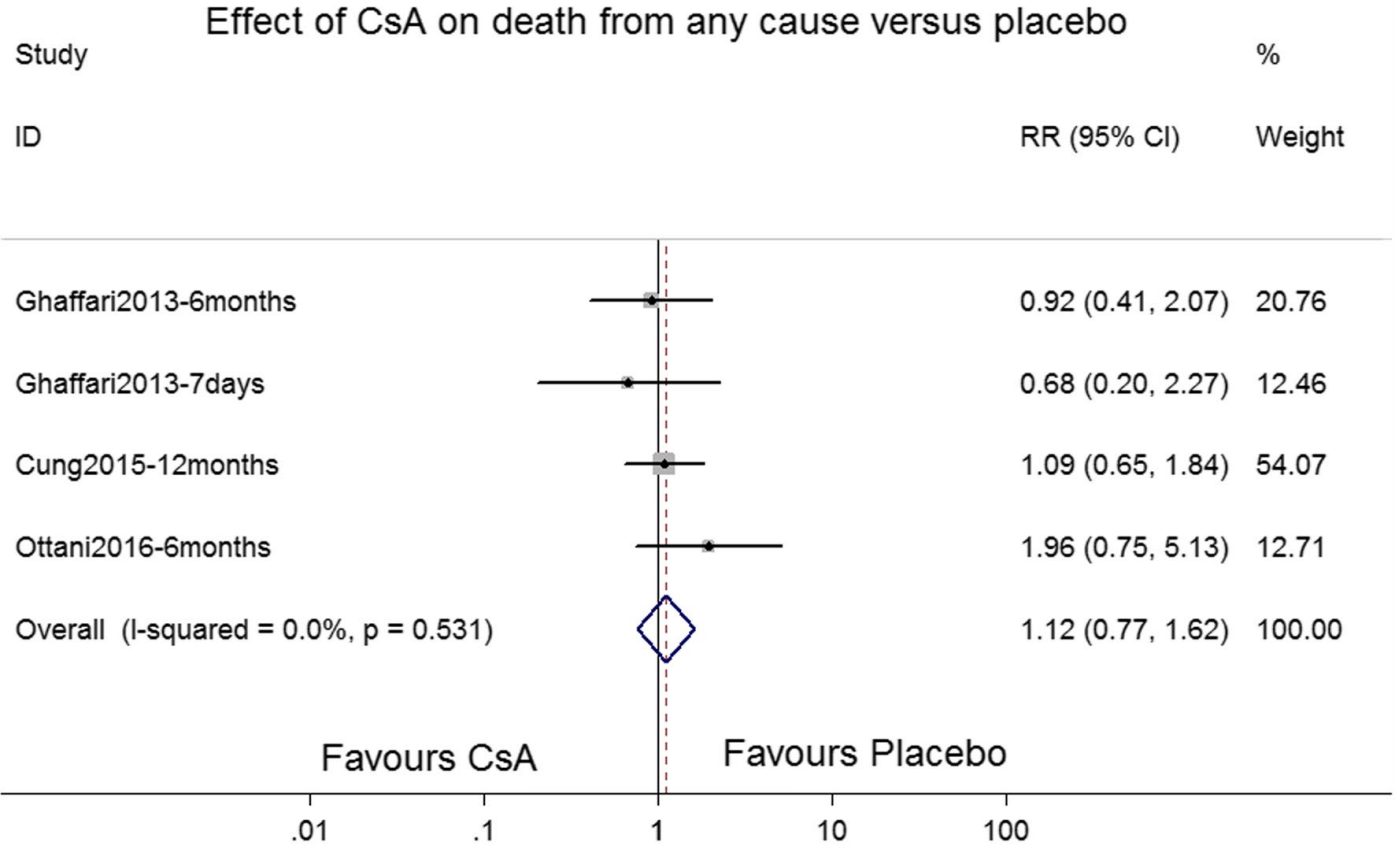
Forest plot depicting the effects of cyclosporine A versus placebo on all-cause mortality rates. *RR* rate ratio, *CI* confidence interval

Three RCTs (Cung et al. 2015; Ghaffari et al. 2013; Ottani et al. 2016) reported the effects of CsA on all-cause mortality compared with those of a placebo. In addition to the in-hospital period, follow-up was performed at 6 and 12 months. The combined data from all RCTs did not show a significant association between CsA treatment and reduced all-cause mortality compared with the placebo (RR 1.10, 95 % CI 0.75–1.61; *P* = 0.533; *I*^2^ = 0 %; Fig. 4).

**Fig. 5:**
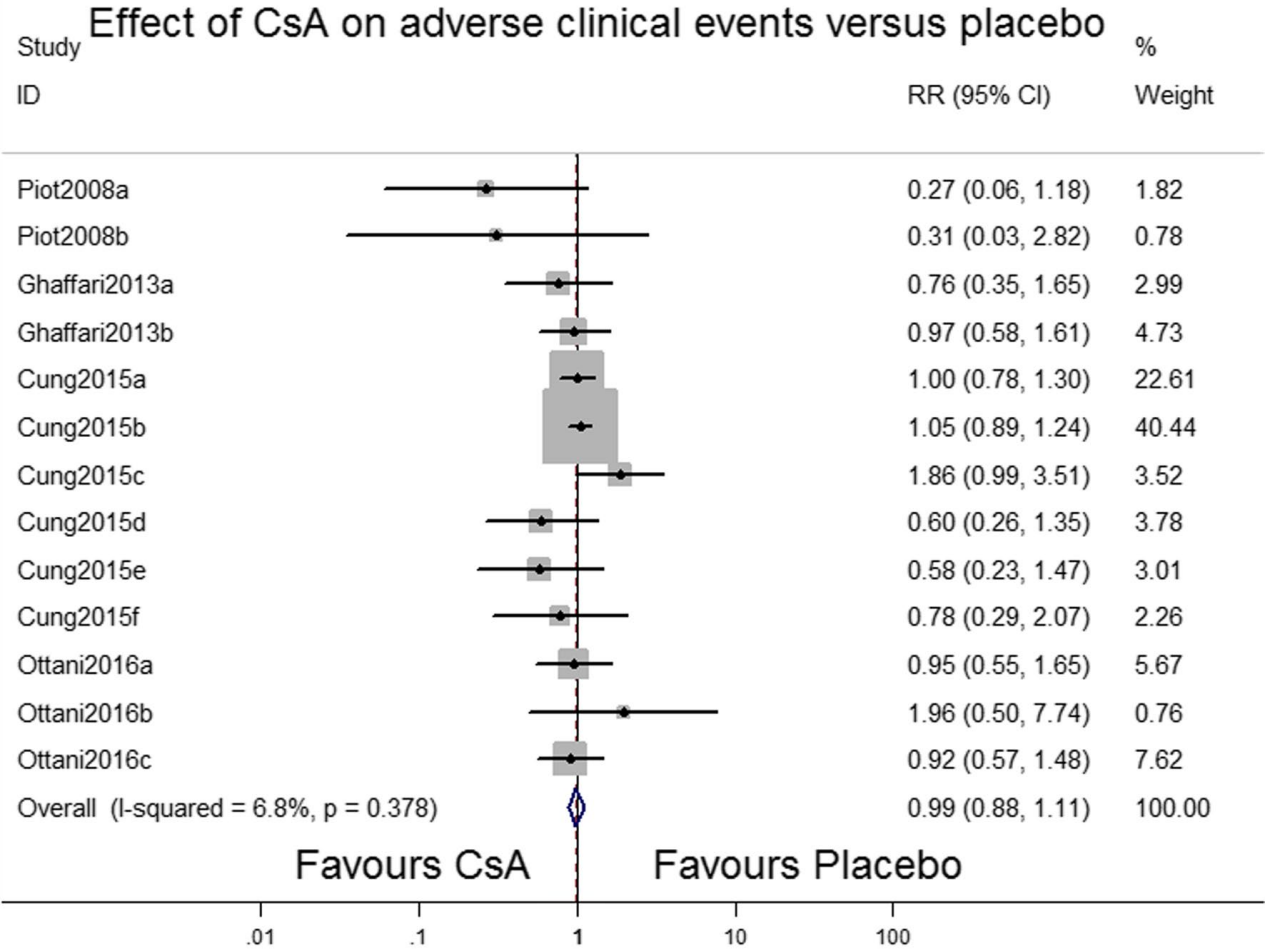
Forest plot depicting the effects of cyclosporine A versus placebo on adverse clinical events. *RR* rate ratio, *CI* confidence interval

Four RCTs (Cung et al. 2015; Ghaffari et al. 2013; Ottani et al. 2016; Piot et al. 2008) have reported the effects of CsA on adverse clinical events including ventricular fibrillation, heart failure, recurrent, ischemia, and bleeding (Table 2). Overall, CsA did not reduce the frequency of adverse clinical events (RR 1.0, 95 % CI 0.89–1.13; *P* = 0.381; *I*^2^ = 6.5 %; Fig. 5).

**Table 2:** Death and adverse clinical event in studies

Study-time	Death or adverse clinical event	Event CsA group event\total	Control group event\total
Piot 2008-2 days	Heart failure	2\30	7\28
Piot 2008-3 months	Heart failure	1\30	3\28
Ghaffari 2012-6 months	Death	9\50	10\51
Ghaffari 2012-7 days	Death	4\50	6\51
Ghaffari 2013-2 days	Major arrhythmia	9\50	12\51
Ghaffari 2013-2 days	Heart failure	18\50	19\51
Cung 2015-12 months	Death	28\475	26\485
Cung 2015-12 months	Heart failure	90\395	90\396
Cung 2015-12 months	Left ventricular remodeling	169\395	161\396
Cung 2015-12 months	Cardiogenic shock	26\395	14\396
Cung 2015-12 months	Recurrent myocardial infarction	9\395	15\396
Cung 2015-12 months	Stroke	7\395	12\396
Cung 2015-12 months	Major bleeding	7\395	9\396
Ottani 2016-6 months	Death	12\207	6\203
Ottani 2016-6 months	Heart failure	21\207	23\203
Ottani 2016-6 months	Cardiogenic shock	6\207	3\203
Ottani 2016-6 months	Re-hospitalization	28\207	30\203

**Fig. 6:**
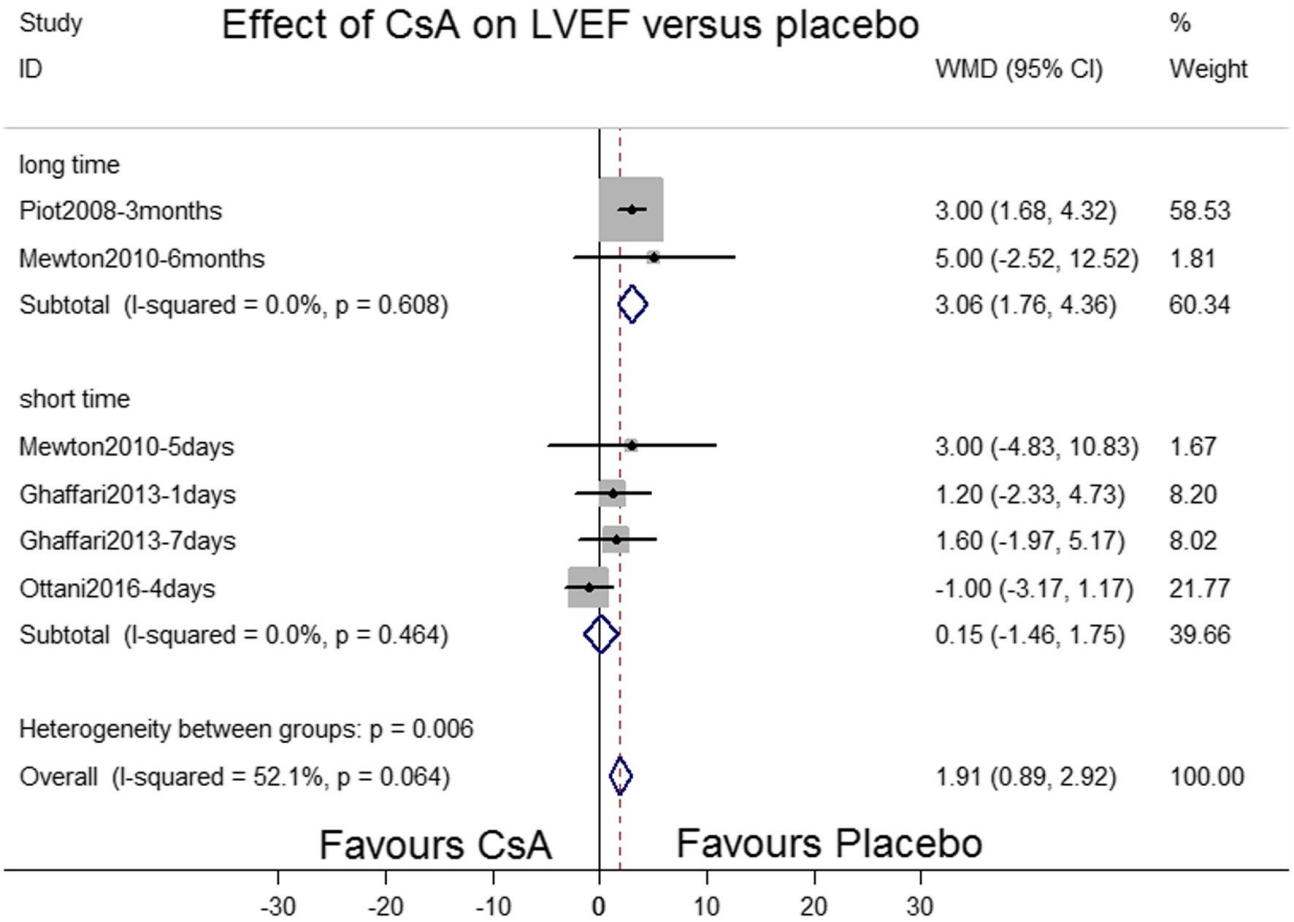
Forest plot depicting the effect of cyclosporine A versus placebo on left ventricular ejection fraction. *WMD* weighted mean difference, *CI* confidence interval

Left ventricular ejection fraction (LVEF) was measured at various stages after MI in four studies (Ghaffari et al. 2013; Mewton et al. 2010; Ottani et al. 2016; Piot et al. 2008): (1) at 5 days and 6 months following MI, (2) at the first day of admission and after hospital discharge, (3) during hospital discharge, and (4) at 4 days after MI. The improvement in the LVEF of the CsA treatment group was minimal compared with that of the placebo group [weighted MD (WMD) = 1.91, 95 % CI 0.89–2.92], with significant heterogeneity (*P* = 0.064; *I*^2^ = 52.1 %). Subgroup analyses were performed for the short-term (<3months) and long-term (>3 months) efficacy of CsA treatment on LVEF. Although no significant improvement was observed in the LVEF of the CsA treatment group compared with that of the placebo group in the short term (WMD = 0.15, 95 % CI −1.46 to 1.75; *P* = 0.464; *I*^2^ = 0.0 %), an increase was noted in the LVEF of the CsA treatment group in the long term (WMD = 3.06, 95 % CI 1.76–4.36; *P* = 0.608; *I*^2^ = 0.0 %; Fig. 6).

**Fig. 7:**
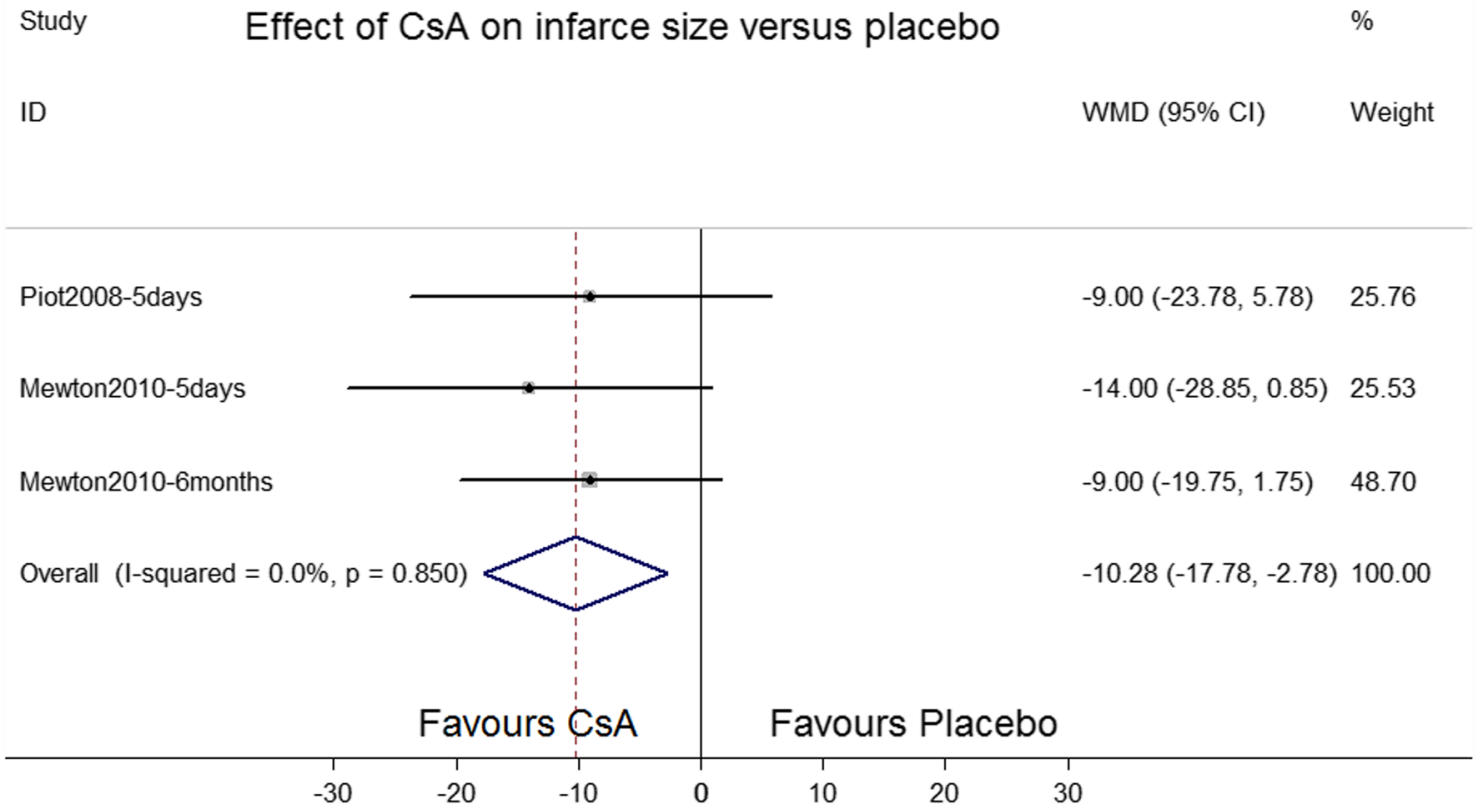
Forest plot depicting the effects of cyclosporine A versus placebo on myocardial infarction size. *SMD* standard mean difference, *CI* confidence interval

MI size was measured through magnetic resonance imaging in one study, twice at 5 days (Piot et al. 2008) and 6 months (Mewton et al. 2010) after MI. A significant reduction in MI size was defined as signal intensity more than 2 standard deviations above that in the reference region of remote noninfarcted myocardium within the same slice. The CsA treatment group appeared to exhibit a smaller reduction in MI size than the placebo group did; however, the difference was nonsignificant (standard MD = −0.41, 95 % CI −0.84 to 0.02; *P* = 0.519; *I*^2^ = 0.0 %; Fig. 7).

### Publication bias

**Fig. 8:**
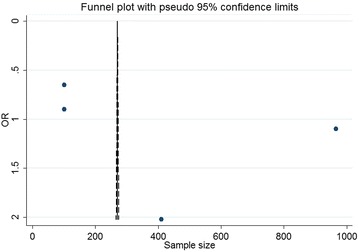
Funnel plot with pseudo 95 % confidence limits depicting the effect of cyclosporine A versus placebo on all-cause mortality

Funnel plots for all-cause mortality rates in the included RCTs for CsA versus a placebo were visually asymmetric (Fig. 8); however, the statistical analysis of these plots suggested an absence of publication bias (*P* = 0.489, Egger’s test). Significant publication biases influenced the effects of CsA on adverse clinical events and LVEF (*P* = 0.022 and 0.004, respectively, Egger’s test).

### Sensitivity analysis

The results of our sensitivity analysis, in which each study was individually excluded from the analysis to evaluate the stability of our estimates, showed no effect on our conclusions.

## Discussion

In our meta-analysis of RCTs involving 1359 patients, CsA did not reduce all-cause mortality or adverse clinical events compared with the placebos used in the investigated studies. However, minimal LVEF improvement and MI size reduction were observed after CsA treatment. In contrast to the positive effects noted in animal models, our results show that CsA administration might not protect the heart from RI in clinical MI patients.

Acute MI (AMI) with early restoration of myocardial perfusion can limit the MI size and improve clinical outcomes. However, reperfusion can result in myocardial damage and limit the benefits of early reperfusion therapies, such as thrombolysis or percutaneous coronary intervention (PCI) after AMI. This phenomenon, termed myocardial RI, may be mediated by a combination of oxidative stress, intracellular Ca^2+^ overload, physiological pH restoration, and neutrophil aggregation (Hausenloy and Yellon 2013).

RI encompasses several distinct pathophysiological components including reversible impaired myocardial contractility (stunning), arrhythmias, no-reflow, and death of cardiomyocytes (lethal RI). Experimental data implicate several factors contributing to lethal RI, independent of no-reflow; such factors include mPTP opening, rapid pH normalization, intracellular calcium overload, and reactive oxygen species generation (Lonborg 2015). A recent meta-analysis (Song et al. 2015) evaluated the effects of CsA for RI on clinical outcomes, including MI size, LVEF, and creatine kinase-MB isoenzyme levels. The results suggested no significant difference between cardiac function and injury with and without CsA treatment; however, the mortality and adverse clinical event data were not reported. In addition, Song et al. (2015) studied the effects of CsA on injury derived both from AMI (primary PCI or thrombolysis) and cardiac surgery (CABG and valve surgery); by contrast, our meta-analysis was specifically focused on AMI. Two relatively larger RCTs regarding the effects of CsA on AMI have been published recently (Cung et al. 2015; Ottani et al. 2016); thus, we conducted this meta-analysis.

CsA is believed to mediate its effect by inhibiting mPTP opening, a major determinant of cell death after ischemic reperfusion. The mPTP is a nonselective pore, the opening of which results in equilibrium between the mitochondria matrix and cytosol, leading to mitochondrial swelling, ATP depletion, and necrotic cell death.

Most studies have investigated specific upstream targets of CsA. However, a pilot study by (Piot et al. 2008) was the first to identify a downstream target of CsA; this can explain the overall neutral results reported in previous studies. Nevertheless, in a large multicenter CIRCUS trial (Cung et al. 2015), intravenous cyclosporine administered to patients with anterior STEMI who were referred for primary PCI attained outcomes similar to those of a placebo, and it did not prevent adverse left ventricular remodeling at 1 year. The CYCLE trial (Ottani et al. 2016) also showed that a single intravenous CsA bolus immediately before primary percutaneous coronary intervention does not affect ST-segment resolution or hs-cTnT, nor does it improve the clinical outcomes or prevent left ventricular remodeling up to 6 months. In our meta-analysis, three studies reported that CsA treatment did not reduce all-cause mortality compared with placebos. In addition, CsA did not prevent adverse clinical events, including ventricular fibrillation, heart failure, recurrent, ischemia, and bleeding. Thus, the results confirmed that CsA does not improve clinical outcomes in reperfused AMI.

MI size is a key indicator for postischemic heart injury and cell death (Burns et al. 2002). Our meta-analysis demonstrates that the MI size of CsA treatment groups does not differ significantly from that of placebo groups, similar to the results reported by a previous meta-analysis (Song et al. 2015). Furthermore, compared with the placebo groups, the CsA treatment groups showed no significant improvement in LVEF during the short-term follow-up, whereas LVEF increased in the placebo groups during long-term follow-up. CsA cardioprotection against RI is effective only when cardiomyocyte apoptosis is highly prevalent (typically during the initial 3–4 h), after which cardiomyocyte necrosis becomes the predominant cardiomyocyte death mechanism; necrosis cannot be prevented by CsA because it is effective only against apoptosis. Moreover, CsA is effective against I/R only when applied during the initial 2–3 h after MI (Lonborg et al. 2012; Santos-Gallego and Badimon 2016). However, the time of CsA administration was inconsistent among the five RCTs in this meta-analysis, extending up to 12 h after chest pain in the study by Cung et al.; therefore, we cannot conclude whether CsA administered in the first 3 h after MI is clinically beneficial.

### Study limitations

Our study has some limitations. First, only five RCTs were included in our meta-analysis. Second, these RCTs had enrolled heterogeneous populations, had different study protocols and endpoint definitions, and had varying follow-up times; these factors might have subjected the results to bias. Finally, significant publication bias for the effect of CsA on adverse clinical events and LVEF was noted; the source of this bias may have been that the indices were drawn from the same study.

## Conclusions

This meta-analysis confirms that CsA may not protect the heart from RI in clinical MI patients. Further research is required to gain additional insight into the nature of RI as a potential therapeutic target.

## Appendix

### Search strategy for EMBASE

“reperfusion”/exp OR reperfusion AND (“cyclosporine”/exp OR cyclosporine) AND myocardial AND (“infarction”/exp OR infarction).

### Search strategy for PubMed

((“reperfusion” [MeSH Terms] OR “reperfusion” [All Fields]) AND (“cyclosporins” [MeSH Terms] OR “cyclosporins” [All Fields] OR “cyclosporine” [All Fields] OR “cyclosporine” [MeSH Terms])) AND (“myocardial infarction” [MeSH Terms] OR (“myocardial” [All Fields] AND “infarction” [All Fields]) OR “myocardial infarction” [All Fields]).
